# Nuclear Translocation of Nuclear Factor Kappa B in First Trimester Deciduas and Chorionic Villi in Early Spontaneous Miscarriage Women

**DOI:** 10.3390/ijms11020521

**Published:** 2010-02-03

**Authors:** Li-qin Wang, Xue-wen Yu, Chun-fang Yan, Xiang Wang

**Affiliations:** 1 Xi’an Medical College, Xi’an, China; E-Mails: wanglq200304@yahoo.cn (L.W.); xiang_w@126.com (X.W.); 2 Center of Maternal and Child Health, First Affiliated Hospital of Medical School in Xi’an Jiaotong University, Xi’an, China; 3 Department of Gynecology & Obstetrics, Fourth Hospital of Xi’an City, Xi’an, China; E-Mail: yanchunfangg@126.com

**Keywords:** early spontaneous miscarriage, nuclear factor kappa B, confocal laser scanning microscope, immunohistochemistry

## Abstract

The nuclear factor kappa B is widely expressed in the distinct subpopulations of chorionic villi and deciduas of first-trimester pregnancies. We examined the cellular distribution and expression of nuclear factor kappa B in the human first-trimester chorionic villi and deciduas of women with early spontaneous miscarriage and viable pregnancy by confocal laser scanning microscope and immunohistochemistry. There is a greater nuclear translocation of nuclear factor kappa B is restricted to villous stromal cells, decidual stromal cells, glandular epithelial cells and vessel endothelial cells in early spontaneous miscarriage than in viable pregnancies. Collectively these observations suggest that over-activation of nuclear factor kappa B has a relationship with early spontaneous miscarriages.

## 1. Introduction

The etiology of spontaneous miscarriage includes chromosomal rearrangements, uterine anomalies, thyroid dysfunction, autoimmune disorders and infection. However, the etiology of approximately 40% of patients is not well known. The intrauterine cytokine balance is an essential condition for normal, successful pregnancy [1,2]. It means that cytokines operating in the embryo and embryonic microenvironment determine, to a significant extent, whether pregnancy is completed successfully or results in embryonic loss or maldevelopment. They act as activators of specific transcription factors, which control cell responses such as cell proliferation differentiation and apoptosis. One such transcription factor is nuclear factor kappa B (NF-κB), which is critically involved in a number of cellular functions, including the cell cycle [3], regulation of apoptosis [4], inflammation [5], aberrant NF-κB activity underling a number of inflammatory diseases and certain cancers [6,7]. In the majority of unstimulated cell types, NF-κB is retained within the cytoplasm in an inactive form, bound to its inhibitor protein, IκB [8]. NF-κB can be activated in at least two ways. One of the ways is that cytokines, such as TNF-α and IL-1β, cause phosphorylation of IκB kinase, ultimately releasing p50:RelA and/or p52/RelA heterodimers [8].

Recently, a number of studies have claimed that TNF-α expression could be detected in human chorionic villi and deciduas of early pregnancy [9]. The TNF-α level has been shown to be significantly elevated in the serum of women with unexplained recurrent spontaneous miscarriage (URSM) [10]. It has also been revealed that mouse blastocysts exposed to TNF-α *in vitro* have an increased death rate when transferred into pseudopregnant mice. These observations have implicated TNF-α as a cytokine involved in triggering immunological pregnancy loss, *i.e.*, death of embryos owing to failure of defense mechanisms preventing rejection of the semiallogeneic fetoplacental unit [11]. In most cell types, TNF-α activates NF-κB through binding to tumor necrosis factor receptor1 (TNFR1) [12]. We recently demonstrated that TNFR1 expression was raised in the decidual cells and the chorionic villous cells of women with early spontaneous miscarriages (ESM), and its increased production correlates with ESM [13,14]. These findings, as well as the properties of TNF-α, gave rise to the hypothesis that TNF-α may activate excessive NF-κB at the feto-maternal interface via TNFR1. NF-κB controls the expression of target genes by binding to their DNA regulatory elements [15,16], and plays a crucial role in regulating apoptotic cell death [17]. Excessive apoptosis in the embryo, regardless of its nature, if uncompensated, ultimately leads to maldevelopment or embryonic death. Consequently, the purpose of the present study was to examine the cellular distribution and expression of NF-κB in the human chorionic villi and deciduas of women with ESM and viable pregnancy, in order to find the relationship between ESM and NF-κB.

## 2. Material and Methods

### 2.1. Subject Population Examined

Thirty-one pregnant women with unexplained vaginal bleeding at 7–10 weeks of gestation, who had been referred to the First Affiliated Hospital of Xi’an Jiaotong University Medical School, Shaanxi Province, China, between August 2004 and October 2005, after confirmed pregnancy loss by ultrasound, were enrolled in the spontaneous miscarriage group (SM group). The diagnosis of normal and failing pregnancies was based on transabdominal and/or transvaginal ultrasound. A gestational sac with no embryo cardiac motion, matched for gestational age, was diagnosed as pregnancy loss, or a gestational sac containing embryo remnants, followed regularly at 1–2 week intervals by ultrasound, that did not develop, was diagnosed as a failing pregnancy. The average age of these women was 29.1 years old and average gestational age was 56.8 days. All of the women examined had a regular menstrual cycle and gestational age, based on the last menstrual period, and confirmed by ultrasound examination. In all of the women chromosomal rearrangements, uterine anomalies, thyroid dysfunction, autoimmune disorders, infection with rubella, toxoplasma, cytomegalovirus and herpes virus, and taken hormone medication in the recent three months, were taken as causes for exclusion from the study. Women (n = 30) without abnormal gynecologic history at 7–10 weeks of viable gestation who wanted to have an induced abortion were included in the control group. Their average age was 27.1 years old and average gestational age was 55.6 days. Live pregnancy was confirmed by ultrasound. All of the women included were in good general health and gave written informed consent before participation. The study was approved by the Department of Science and Research of the First Affiliated Hospital of Xi’an Jiaotong University Medical School.

### 2.2. Measurement of Chorionic Villous and Decidual P65 Component of NF-κB by Confocal Laser Scanning Microscopy

Chorionic villous and decidual samples were collected by curettage from women with SM [31] and viable pregnancy [30] undergoing induced abortion. All samples were washed in 0.9% NaCl as soon as the chorionic villi and deciduas had been removed from the uterus. After fixing in 4% buffered polyformaldehyde for 6 hours at room temperature, the samples were stored in 25% saccharose solution overnight at 4 °C, and then frozen in a −70 °C refrigerator. Sections (10-μm and 5-μm-thick) were prepared by means of a tissue microslicer (Microm HM500 O, Germany). At least one section from each case was stained with haematoxylin and eosin (H&E) to allow morphological assessment. If cellular degeneration and necrosis were observed, the case was removed from the study.

The fluorescence intensity corresponding to the value of NF-κB protein p65 was measured employing a CLSM (TCS SP2, Leica Co., Germany), in accordance with the manufacturer’s instructions. Briefly, after air drying, the 10-μm-thick sections were immersed in a buffer containing 30% H_2_O_2_ and distilled water (30% H_2_O_2_:distilled water = 1:10) for 5 min at room temperature. Then, after rinsing in distilled water three times (5 min each), the sections were sealed in a buffer containing normal bovine serum albumin at a concentration of 1 mg/mL for 20 min at room temperature, followed by incubation with 50 μL of rabbit anti-human NF-κB protein p65 antibody diluted at 1:25 (Santa-Cruz Biotechnology, Inc., USA) for 2 hours at 37 °C. Fluorolabeling was carried out by reacting sections for 30 min with FITC-conjugated goat anti-rabbit IgG antibody (Southern Biology, USA) at 37 °C. They were then rinsed three times in PBS buffer, pH 7.4, for 5 min each time and mounted on glass slides with the aid of glycerol buffer. The P65 fluorescence distribution was observed using the CLSM. The fluorescence excitation was provided by a 488 nm argon laser beam and emission was 543 nm. Five locations were scanned for each sample by CLSM. The images were analyzed using the Image Plus software (Leica, Germany). The mean fluorescence intensity of positive cells was analyzed within the cell population.

### 2.3. Assessment of Chorionic Villous and Decidual P65 Component of NF-κB by SP Immunohistochemistry

Five μm-thick sections frozen from 31 SM and 30 control villous samples were analyzed with a SP immunohistochemistry kit (Zymed Laboratories, Inc., USA). Briefly, after sealing in buffer containing normal bovine serum albumin and incubation with 50 μL of rabbit anti-human anti-NF-κB protein p65 antibody as mentioned above, the sections were incubated with biotinylated goat anti-rabbit IgG for 30 min. For the negative control, the primary antibody was omitted. Sections were then incubated in DAB reagent and counterstained with hematoxylin and coverslipped using Protexx mounting media (DAB-Stock Stain box; Wuhan Boster Biotechnology Co., China). Cells (n = 1,000) were counted in a blinded fashion by two independent pathologists. A positive reaction for NF-κB protein p65 was defined as a brown-yellow granulation in the cellular membrane and cytoplasm. Positive classified criteria of cyto-chromatism were determined according to Garcia’s method [18], *i.e.*, hypo-yellow granulation of the cellular membrane and cytoplasm equals 1, brown-yellow as 2, and hyper-yellow as 3. Villus or decidual samples was defined as having a score of 1 when less than 20% of cells on one slide had cellular membrane and cytoplasmic staining, 2 between 20%–50% of cells, and 3 more than 50% of cells were stained. When these two parameters, as described above, were measured for each cell and when their scores were added together, a score of 2–3 was considered a weak-positive (+), 3–4 as a positive (++), and ≥4 to be a strong positive (+++). Negative cells had clear cell structure without brown granulation in their cellular membrane and cytoplasm. For statistical analysis, the SPSS-PC+ software was used. Intensity of fluorescence was expressed as the mean ± SE. Statistical analysis was performed using Student’s t-test to statistically compare the intensity of fluorescence of villous cells and decidual cells from control group and SM group. Fisher’s Exact test was performed to compare the descriptive data. A value of P < 0.05 was considered significant.

## 3. Results

Human chorionic villous cells and decidual cells were observed with by CLSM and SP immunohistochemistry. Compared the SM group with the control group, there was no significant difference with respect to the subjects’ age and the gestational age.

### 3.1. Immunolocalization of NF-κB Protein P65 in the Human Chorionic Villi and Deciduas

**Figure 1.**
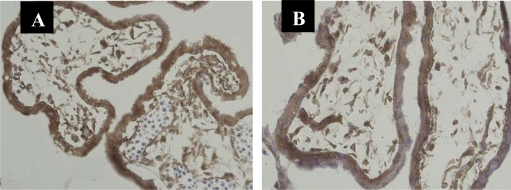
Significantly lower staining score with NF-κB in villous syncytiotrophoblasts in the SM group than in the control group by immunohistochemistry. (A), Control; (B), Early spontaneous miscarriage. SP × 200.

**Figure 2.**
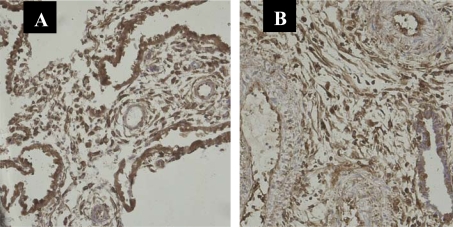
Significantly higher staining score with NF-κB in decidual stromal cells and lower staining score with NF-κB in decidual glandular epithelial cells in the SM group than in the control group by immunohistochemistry. (A) Control; (B) Early spontaneous miscarriage. SP × 200.

All cases showed NF-κB protein p65 positive staining immunolocalized to the cytoplasm and nucleus of first-trimester chorionic villous and decidual cells in both SM and control groups. These cells included villous stromal cells, vessel endothelial cells, cytotrophoblasts and syncytiotrophoblasts of first-trimester chorionic villi, and decidual glandular epithelial cells, stromal cells and vessel endothelial cells of first-trimester decidual tissues. The important observations from the statistical analysis of the data can be summarized as follows: (i) significantly lower staining score with NF-κB in villous syncytiotrophoblasts in the SM group than in the control group (p < 0.001; Figure 1, Table 1); (ii) significantly higher staining score with NF-κB in decidual stromal cells and lower staining score with NF-κB in decidual glandular epithelial cells in the SM group than in the control group (p < 0.001; Figure 2, Table 2). We failed to detect significant expressive difference with NF-κB in the villous stromal cells, vessel endothelial cells, cytotrophoblasts, and decidual vessel endothelial cells of spontaneous miscarriages, compared to the viable pregnancies.

### 3.2. Nuclear Translocation of the NF-κB Protein P65 in the Human Chorionic Villi

**Figure 3.**
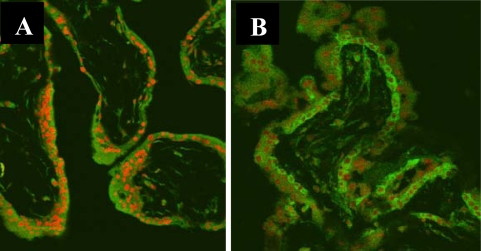
Immunofluorescence in the unclei and nuclear translocation with NF-κB protein p65 in villi. (A), Control; (B), Early spontaneous miscarriage. CLSM × 200 (Green color indicates positive staining for NF-κB protein p65; Red color indicates nuclei).

Human chorionic villous cells including villous stromal cells, vessel endothelial cells, syncytiotrophoblast cells and cytotrophoblast cells in both SM and control groups were observed. Since it is amply documented that localization of NF-κB in the nucleus is evidence for its activation, we took an immunofluorescence approach using p65 antibody with a CLSM. Immunofluorescence was observed in the cytoplasm and nuclei of villous stromal, vessel endothelial, syncytiotrophoblast and cytotrophoblast cells in both SM and control groups. However, the fluorescence intensity in the nuclei varied among the villous cells in both SM and control groups. The nuclei of stromal cells showed strong fluorescence intensity in SM and but appeared weak intensity in the control group (Figure 3). A similar immunofluorescence of p65 was observed among the vessel endothelial, syncytiotrophoblast and cytotrophoblast cells between SM and control groups. The level of the nucleoplasmic ratio of NF-κB p65 (mean ± s) in the different cells was shown in Table 3. The nucleoplasmic ratio of NF-κB p65 in villous stromal cells was significantly higher in SM group than in the control group, and no significant difference for the ratio was observed in the villous vessel endothelial cells, syncytiotrophoblast cells and cytotrophoblast cells in SM, compared to the control group. The same results were found comparing women with first SM with viable pregnancies, indicating that NF-κB is cell-specifically activated in nonviable pregnancies.

### 3.3. Nuclear Translocation of the NF-κB Protein P65 in the Human Deciduas

**Figure 4.**
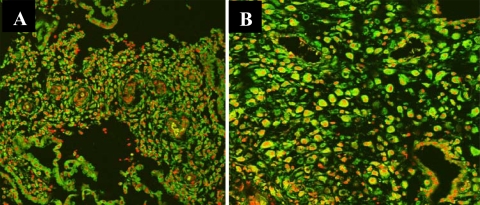
Immunofluorescence in the cytoplasm and nuclear translocation with NF-κB protein p65 in deciduas. (A), Control; (B), Early spontaneous miscarriage. CLSM × 200 (Green color indicates positive staining for NF-κB protein p65; Red color indicates nuclei).

The glandular epithelial cells, stromal cells and vessel endothelial cells in deciduas were observed. The p65 component of NF-κB was expressed in the three types of cells. The weak immunofluorescence in the cytoplasm of decidual glandular epithelial, stromal and vessel endothelial cells in SM was observed and but only had statistical significance in stromal cells (P < 0.05; Figure 4), compared to the control group. The nucleoplasmic ratio of NF-κB p65 (mean ± s) in the three types of cells was significantly higher in SM than that in the control group (P < 0.001, Table 4). There was the similarly nucleoplasmic ratio of NF-κB p65 among decidual glandular epithelial cells, stromal cells and vessel endothelial cells.

These results indicate that NF-κB is activated specifically at the decidual stromal cells, glandular epithelial cells and vessel endothelial cells from women with SM.

## 4. Discussion

NF-κB protein p65 expression was detected in the human first-trimester chorionic villous and decidual tissues. NF-κB was widely expressed in villous and decidual cells of first-trimester pregnancies. In the chorionic villous tissue, NF-κB was found to be weakly expressed in villous syncytiotrophoblast in the early spontaneous miscarriages. In the decidual tissue, NF-κB was intensely expressed in decidual stromal cells, whereas weakly expressed in decidual glandular epithelial cells in the early spontaneous miscarriages. Collectively, these observations strengthen our hypothesis that NF-κB has relationship with the early spontaneous miscarriage. Aban, *et al*. have found increased NF-κB expression in placental trophoblastic epithelium from IUGR and preeclampsia and speculated that placental trophoblastic apoptosis might have resulted from NF-κB dependent pathway [19]. NF-κB may play key regulatory roles in human success pregnancy by eliciting their biological actions on the subpopulations of trophoblasts and decidual cells present at the maternal-fetal interface.

In the study, we also showed the nuclear translocation of the p65 component of NF-κB by the human early chorionic villi and deciduas *in vivo*. Our findings illustrate that there is a greater nuclear translocation of the NF-κB protein p65 in chorionic villi and deciduas from women with early spontaneous miscarriage, but a significant increase was observed in villous stromal cells, decidual glandular epithelial cells, decidual stromal cells and decidual vessel endothelial cells compared to viable pregnancies. The p65 is only one component of the NF-κB family. This is the first study showing expression of these proteins in the human early chorionic villi and deciduas and needs to be confirmed by others. NF-κB is presently seen as a critical regulator of the development and maintenance of the immune system. NF-κB also is a family of transcription factors involved in the control of genes coding for cytokines and adhesion molecules [5]. It is important in mediating the action between IL-1 and TNF-α receptors and IL-6 and LIF production by human BeWo cells and human endometrial epithelial cells [20,21]. In human term and preterm placenta, amnion, and choriodecidua, NF-κB activation promotes the formation of IL-6, IL-8, TNF-α and the anti-inflammatory agent sulfasalazine concentrations greater than 5 mM, through its ability to inhibit NF-κB activation, inhibits release of IL-2, IL-8, and TNF-α [22]. Cytokine induced NF-κB can precipitate a positive feedforward loop resulting in amplification of cytokine production and further NF-κB activation. Others have suggested that NF-κB may be involved in the inflammatory events associated with menstruation [23,24], and the increased production of pro-inflammatory cytokines lead to production of matrix metalloproteinases (MMPs) which are involved in tissue breakdown. McCracken *et al*. [25] reported that NF-κB down regulation in T cells of pregnant women is essential for maintaining the cytokine profile necessary for pregnancy success. Suppression of NF-κB activation in first trimester deciduas has also been suggested to contribute to the immunosuppressive mechanisms that prevail during pregnancy [23]. However, if the pattern of expression is confirmed, this study together with our previous study [13] suggests that at the first trimester placenta with nonviable pregnancy there is increased expression of the active components of the NF-κB family of proteins and TNFR1, further suggesting their importance in the control of factors involved in pregnancy success. Still, it is not clear that whether NF-κB forced increased mTNFR1 expression or elevated mTNFR1 expression forced the activation of NF-κB. To this end, further research is necessary to fully understand the relationship between NF-κB and mTNFR1 in maternal-fetal interface. However, the development of specific inhibitors of NF-κB will be both beneficial in dissecting the role of NF-κB in the initiation of spontaneous miscarriage and could potentially be clinically useful in the management and treatment of miscarriage. Others have already found that 17β-estradiol at a physiologically high concentration modulates NF-κB signaling in human T cells and affects T cell survival [26].

The present study has demonstrated differential nuclear translocation of NF-κB by subpopulations of chorionic villous cells in early pregnancies. The nucleoplasmic ratio of NF-κB in villous stromal cells is more in women with SM than those in viable pregnancy. It means that NF-κB is activated in villous stromal cells with the nonviable pregnancy. The elevated mTNFR1 in the first trimester placenta in nonviable pregnancies is now also shown in placental villi, predominantly in stromal cells [14]. In view of evidence above, it may be speculated that up-regulated local mTNFR1 in villous stromal cells stimulates the activation of NF-κB in nonviable pregnancies (this needs to be demonstrated further). It could possibly cause pathological changes or tissue damage in the chorionic villi locally. To data, immunostaining indicates that a subpopulation of placental villous mesenchymal cells produces insulin-like growth factor [27]. Moreover, fetal growth is dependent on insulin-like growth factor in humans [28], and the progesterone receptor is detectable in villous stromal cells. Progesterone plays pivotal roles during the implantation process and is essential in maintaining an ongoing pregnancy [29]. Therefore, it is important to keep the balance of cytokine profile in chorionic villous stromal cells for pregnancy success. One more insufficiency is that the approximate length of time from fetal demise to tissue collection could not be ascertained due to lack of constant ultrasonography examination. The relationship between the results obtained and the fetal demise can not be exactly defined. It is possible that the results obtained are a reflection of an inflammatory response to and causative of it, which needs further research.

## 5. Conclusions

In summary, we have determined that NF-κB is widely expressed among the distinct subpopulations of chorionic villi and deciduas of the first-trimester placenta, whereas the over activation of NF-κB is restricted to villous stromal cells, decidual stromal cells, glandular epithelial cells and vessel endothelial cells in nonviable pregnancies. Collectively these observations suggest that NF-κB is capable of causing pathological changes or tissue damage within the tissues in nonviable pregnancies. The control of NF-κB activation may therefore provide an alternative therapeutic strategy for reducing the release of pro-inflammatory mediators in early spontaneous miscarriages.

## Figures and Tables

**Table 1.** Staining score with NF-κB in villous syncytiotrophoblasts.

	−	+	++	+++
SM group	4	16	9	2
Control group	0	4	14	12
P*			<0.001	

**Table 2.** Staining score with NF-κB in decidual stromal cells and decidual glandular epithelial cells.

	**stromal cells**				**Glandular epithelial cells**			
	−	+	++	+++	−	+	++	+++
**SM group**	0	0	2	29	2	14	9	6
**Control group**	0	5	9	16	0	2	10	8
P*			0.001				<0.001	

− = completely absent; + = trace amount; ++ = moderate amount; +++ = appreciable amount.

* Fisher’s Exact Test.

**Table 3.** The level of the nucleoplasmic ratio of NF-κB p65 (mean ± s) in the different cells in villous.

	**stromal cells**	**vessel endothelial cells**	**syncytiotrophoblast cells**	**cytotrophoblast cells**
**SM group**	0.7602 ± 0.0775	0.7191 ± 0.0516	0.6632 ± 0.0564	0.7042 ± 0.0619
**Control group**	0.6977 ± 0.0636	0.6896 ± 0.0705	0.6336 ± 0.0823	0.6701 ± 0.0778
**T**	3.511	1.514	1.741	1.935
**P**	0.001	0.135	0.086	0.057

**Table 4.** The level of the nucleoplasmic ratio of NF-κB p65 (mean ± s) in the different cells in deciduas.

	**glandular epithelial cells**	**stromal cells**	**Vessel endothelial cells**
**SM group**	0.7184 ± 0.0553	0.8235 ± 0.0665	0.7484 ± 0.0700
**Control group**	0.6484 ± 0.0642	0.5840 ± 0.0503	0.6625 ± 0.0576
**T**	4.422	15.201	5.029
**P**	<0.001	<0.001	<0.001
